# Study in Parkinson’s disease of exercise phase 3 (SPARX3): study protocol for a randomized controlled trial

**DOI:** 10.1186/s13063-022-06703-0

**Published:** 2022-10-06

**Authors:** Charity G. Patterson, Elizabeth Joslin, Alexandra B. Gil, Wendy Spigle, Todd Nemet, Lana Chahine, Cory L. Christiansen, Ed Melanson, Wendy M. Kohrt, Martina Mancini, Deborah Josbeno, Katherine Balfany, Garett Griffith, Mac Kenzie Dunlap, Guillaume Lamotte, Erin Suttman, Danielle Larson, Chantale Branson, Kathleen E. McKee, Li Goelz, Cynthia Poon, Barbara Tilley, Un Jung Kang, Malú Gámez Tansey, Nijee Luthra, Caroline M. Tanner, Jacob M. Haus, Giamila Fantuzzi, Nikolaus R. McFarland, Paulina Gonzalez-Latapi, Tatiana Foroud, Robert Motl, Michael A. Schwarzschild, Tanya Simuni, Kenneth Marek, Anna Naito, Codrin Lungu, Daniel M. Corcos, The SPARX3-PSG Investigators, Terry D. Ellis, Ludy C. Shih, Timothy J. Nordahl, Michael T. Stevenson, Jay L. Alberts, Ashwini K. Rao, Corey Landis, Joe R. Nocera, Madeleine E. Hackney, Elizabeth L. Stegemoller, Angela L. Ridgel, Jan M. Hondzinski, Neil M. Johannsen, Patrick Drummond, Heather Milton, David A. Hinkle, Fay B. Horak, Mitra Afshari, Christopher P. Hurt, Ariel Kidwell, Corinna Conroy, Neil Panchal, Brooke Schultz, Jes Marchbank, Aaron Bloemer, Demetra D. Christou, David E. Vaillancourt, Stephanie Lapierre, Colum D. MacKinnon, Sommer Amundsen-Huffmaster, Kristin Garland, Blake B. Rasmussen, Summer Chapman, Jessica Spahn, Laura Wu, Lee E. Dibble, Genevieve N. Olivier, Art Weltman, William Alex Dalrymple, David Edwards, Corey Rynders, Lauren Miller, Gammon M. Earhart, Kerri S. Rawson, Kelvin Jones, Krista Nelles, Quincy J. Almeida, Marie Saint-Hilaire, Stewart A. Factor, Camilla Kilbane, Brian J. Copeland, Marian L. Dale, Alberto J. Espay, Adolfo Ramirez-Zamora, Amanda Fessenden, Andres F. Deik, Richard Camicioli

**Affiliations:** 1 Department of Physical Therapy, University of Pittsburgh, School of Health and Rehabilitation Sciences, 100 Technology Drive, Suite 500, Pittsburgh, PA 15219 USA; 2 Department of Physical Therapy and Human Science, Northwestern University, Feinberg School of Medicine, Suite 1100, 645 North Michigan Avenue, Chicago, IL 60305 USA; 3 Department of Neurology, University of Pittsburgh, School of Medicine, 3471 Fifth Avenue, Pittsburgh, PA 15213 USA; 4 Department of Physical Medicine & Rehabilitation, University of Colorado, School of Medicine, Aurora, CO 80217 USA; 5 Division of Endocrinology, Metabolism and Diabetes, and Division of Geriatric Medicine, Department of Medicine, University of Colorado Anschutz Medical Campus, Aurora, CO USA; 6 Eastern Colorado VA Health Care System, Geriatric Research Education and Clinical Center (GRECC), Denver, CO USA; 7 Division of Geriatric Medicine, Department of Medicine, University of Colorado Anschutz Medical Campus, Aurora, CO USA; 8 Eastern Colorado Geriatric Research, Education, and Clinical Center, Rocky Mountain Regional VAMC, Aurora, USA; 9 Department of Neurology, Oregon Health & Science University, 3181 SW Sam Jackson Road, Portland, OR 97219 USA; 10 Neurological Institute, Cleveland Clinic, 9500 Euclid Ave, Cleveland, OH 44195 USA; 11 Movement Disorders Division, Department of Neurology, University of Utah, 175 Medical Dr N, Salt Lake City, UT 84132 USA; 12 Department of Physical Therapy & Athletic Training, University of Utah, 520 Wakara Way, Salt Lake City, UT 84115 USA; 13 Department of Neurology, Feinberg School of Medicine, Northwestern University, Suite 115, 710 N Lake Shore Drive, Chicago, IL 60611 USA; 14 Morehouse School of Medicine, 720 Westview Dr SW, Atlanta, GA 30310 USA; 15 Neurosciences Clinical Program, Intermountain Healthcare, 5171 S Cottonwood Street, Suite 810, Murray, UT 84107 USA; 16 Department of Kinesiology and Nutrition, UIC College of Applied Health Sciences, 919 W Taylor Street, Chicago, IL 60612 USA; 17 Department of Biostatistics and Data Science, University of Texas Health Science Center School of Public Health, 1200 Pressler Street E835, Houston, TX 77030 USA; 18 NYU Langone Health, NYU Grossman School of Medicine, 435 E 30th Street, Science Building 1305, New York, NY 10016 USA; 19 Department of Neuroscience and Neurology, Normal Fixel Institute for Neurological Diseases and College of Medicine, University of Florida, 4911 Newell Road, Gainesville, FL 32610 USA; 20 Department of Neurology, Weill Institute for Neurosciences, University of California San Francisco, 1651 4th Street, San Francisco, CA 94158 USA; 21 School of Kinesiology, University of Michigan, 830 N. University Ave, Ann Arbor, MI 48109 USA; 22 Department of Neurology, Norman Fixel Institute for Neurological Diseases, College of Medicine, University of Florida, Gainesville, FL 32608 USA; 23 Department of Medical and Molecular Genetics, Indiana University School of Medicine, 410 W. 10th Street, Indianapolis, IN 46220 USA; 24 Mass General Institute for Neurodegenerative Disease, Massachusetts General Hospital, Rm 3002, 114 16th Street, Boston, MA 02129 USA; 25 Institute for Neurodegenerative Disorders, 60 Temple St, New Haven, CT 06510 USA; 26 Parkinson’s Foundation 200 SE 1st Street Suite 800, Miami, FL 33131 USA; 27 National Institute of Neurological Disorders and Stroke, NIH, 6001 Executive Blvd, #2188, Rockville, MD 20852 USA

**Keywords:** Parkinson disease, Endurance exercise, Treadmill exercise, Exercise dose response, DaTscan™ SPECT, Gait assessment, Quality of life, Time to initiate dopaminergic medication, Blood biomarkers

## Abstract

**Background:**

To date, no medication has slowed the progression of Parkinson’s disease (PD). Preclinical, epidemiological, and experimental data on humans all support many benefits of endurance exercise among persons with PD. The key question is whether there is a definitive additional benefit of exercising at high intensity, in terms of slowing disease progression, beyond the well-documented benefit of endurance training on a treadmill for fitness, gait, and functional mobility. This study will determine the efficacy of high-intensity endurance exercise as first-line therapy for persons diagnosed with PD within 3 years, and untreated with symptomatic therapy at baseline.

**Methods:**

This is a multicenter, randomized, evaluator-blinded study of endurance exercise training. The exercise intervention will be delivered by treadmill at 2 doses over 18 months: moderate intensity (4 days/week for 30 min per session at 60–65% maximum heart rate) and high intensity (4 days/week for 30 min per session at 80–85% maximum heart rate). We will randomize 370 participants and follow them at multiple time points for 24 months. The primary outcome is the Movement Disorders Society-Unified Parkinson’s Disease Rating Scale (MDS-UPDRS) motor score (Part III) with the primary analysis assessing the change in MDS-UPDRS motor score (Part III) over 12 months, or until initiation of symptomatic antiparkinsonian treatment if before 12 months. Secondary outcomes are striatal dopamine transporter binding, 6-min walk distance, number of daily steps, cognitive function, physical fitness, quality of life, time to initiate dopaminergic medication, circulating levels of C-reactive protein (CRP), and brain-derived neurotrophic factor (BDNF). Tertiary outcomes are walking stride length and turning velocity.

**Discussion:**

SPARX3 is a Phase 3 clinical trial designed to determine the efficacy of high-intensity, endurance treadmill exercise to slow the progression of PD as measured by the MDS-UPDRS motor score. Establishing whether high-intensity endurance treadmill exercise can slow the progression of PD would mark a significant breakthrough in treating PD. It would have a meaningful impact on the quality of life of people with PD, their caregivers and public health.

**Trial registration:**

ClinicalTrials.govNCT04284436. Registered on February 25, 2020.

## Administrative information

Note: the numbers in curly brackets in this protocol refer to SPIRIT checklist item numbers. The order of the items has been modified to group similar items (see http://www.equator-network.org/reporting-guidelines/spirit-2013-statement-defining-standard-protocol-items-for-clinical-trials/).

**Table:** 

Title {1}	Study in Parkinson Disease of Exercise Phase 3 Randomized Clinical Trial (SPARX3)
Trial registration {2a and 2b}.	NCT04284436, ClinicalTrials.gov https://clinicaltrials.gov/ct2/show/NCT04284436 First Posted: February 25, 2020
Protocol version {3}	Version Number 1.7 10/18/2021
Funding {4}	National Institute of Neurological Disease and Stroke of the National Institutes of Health, U01 NS113851-01
Author details {5a}	1. Department of Physical Therapy, University of Pittsburgh, School of Health and Rehabilitation Sciences, 100 Technology Drive, Suite 500, Pittsburgh, PA, 15219, USA 2. Department of Physical Therapy and Human Science, Northwestern University, Feinberg School of Medicine, Suite 1100, 645 North Michigan Avenue, Chicago, IL, 60305, USA 3. Department of Neurology, University of Pittsburgh, School of Medicine, 3471 Fifth Avenue, Pittsburgh, PA, 15213, USA. 4. Department of Physical Medicine & Rehabilitation, University of Colorado, School of Medicine, Aurora, CO, 80217, USA 5. Division of Endocrinology, Metabolism and Diabetes, and Division of Geriatric Medicine, Department of Medicine, University of Colorado Anschutz Medical Campus, Aurora, CO, USA 6. Eastern Colorado VA Health Care System, Geriatric Research Education and Clinical Center (GRECC), Denver, CO, USA 7. Division of Geriatric Medicine, Department of Medicine, University of Colorado Anschutz Medical Campus, Aurora, CO, USA 8. Eastern Colorado Geriatric Research, Education, and Clinical Center, Rocky Mountain Regional VAMC, Aurora, USA 9. Department of Neurology, Oregon Health & Science University, 3181 SW Sam Jackson Road, Portland, OR, 97219, USA 10. Neurological Institute, Cleveland Clinic, 9500 Euclid Ave, Cleveland, OH, 44195, USA 11. Movement Disorders Division, Department of Neurology, University of Utah, 175 Medical Dr N, Salt Lake City, UT, 84132, USA 12. Department of Physical Therapy & Athletic Training, University of Utah, 520 Wakara Way, Salt Lake City, UT, 84115, USA 13. Department of Neurology, Feinberg School of Medicine, Northwestern University, Suite 115, 710 N Lake Shore Drive, Chicago, IL, 60611, USA 14. Morehouse School of Medicine, 720 Westview Dr SW, Atlanta, GA, 30310, USA 15. Neurosciences Clinical Program, Intermountain Healthcare, 5171 S Cottonwood Street, Suite 810, Murray, UT, 84107, USA 16. Department of Kinesiology and Nutrition, UIC College of Applied Health Sciences, 919 W Taylor Street, Chicago, IL, 60612, USA 17. Department of Biostatistics and Data Science, University of Texas Health Science Center School of Public Health, 1200 Pressler Street E835, Houston, TX, 77030, USA 18. NYU Langone Health, NYU Grossman School of Medicine, 435 E 30th Street, Science Building 1305, New York, NY, 10016, USA 19. Department of Neuroscience and Neurology, Normal Fixel Institute for Neurological Diseases and College of Medicine, University of Florida, 4911 Newell Road, Gainesville, FL, 32610, USA 20. Department of Neurology, Weill Institute for Neurosciences, University of California San Francisco, 1651 4th Street, San Francisco, CA, 94158, USA 21. School of Kinesiology, University of Michigan, 830 N. University Ave, Ann Arbor, MI, 48109, USA 22. Department of Neurology, Norman Fixel Institute for Neurological Diseases, College of Medicine, University of Florida, Gainesville, FL, 32608, USA 23. Department of Medical and Molecular Genetics, Indiana University School of Medicine, 410 W. 10th Street, Indianapolis, IN, 46220, USA 24. Mass General Institute for Neurodegenerative Disease, Massachusetts General Hospital, Rm 3002, 114 16th Street, Boston, MA, 02129, USA 25. Institute for Neurodegenerative Disorders, 60 Temple St, New Haven, CT, 06510, USA 26. Parkinson’s Foundation 200 SE 1st Street Suite 800, Miami, FL, 33131, USA 27. National Institute of Neurological Disorders and Stroke, NIH, 6001 Executive Blvd, #2188, Rockville, MD, 20852, USA
Name and contact information for the trial sponsor {5b}	NINDS - Neuroscience Center Division of Extramural Activities 6001 Executive Boulevard Suite 3309 Bethesda, MD 20892- 9531
Role of sponsor {5c}	The sponsor provided input into the design to facilitate the extension of follow-up from 12 months to 24 months. The sponsor required use of NINDS Common Data Elements for data collection. The sponsor has no role in the management, analysis, or interpretation of the data; writing of this report; and the decision to submit the report for publication.

## Introduction

### Background and rationale {6a}

Resistance exercise, balance exercise, and endurance exercise are therapeutically beneficial for people with Parkinson’s disease (PD) [1, 2, 3, 4]. Despite being proposed as the “Universal prescription for Parkinson’s disease” [5], most clinicians who treat people with PD are still unclear about how best to prescribe endurance exercise. In contrast, medications for PD are prescribed with specified doses and frequencies due to large scientific investigations demonstrating efficacy and side effects [6]. Exercise has great clinical potential given the ease of implementation, safety, and physiological benefits [7]. However, exercise regimens in PD have not undergone the pipeline of testing in Phase 2 and Phase 3 trials, unlike their pharmacologic counterparts, leaving many unanswered questions about dosing and efficacy. This necessarily limits confidence in clinical recommendations.

Exercise has been shown to regulate brain function [8, 9, 10, 11] and modify the signs and symptoms of PD. [12] There is mounting evidence that it also protects against neurological damage in animal models [13]. Several principles have emerged for modifying the symptoms of neurological insult through exercise: specificity of training is important, for example the best way to improve walking is by walking, repetition is critical, and exercise intensity matters. These principles have been applied to animal models of PD with attempts to reduce the parkinsonian symptoms resulting from neurochemical damage [14, 15, 16] with emphasis on skill development [15] and gait training [14, 16, 17]. The mechanisms by which exercise modifies brain function are not well understood but could include increased cerebral blood flow [8], increased cerebral vascular reactivity [18], increased production of anti-inflammatory cytokines [19, 20, 21], and increased brain-derived neurotrophic factor (BDNF) [22]. A possible neuroprotective effect on striatal (GABAergic) medium spiny neurons has also been demonstrated in toxin-based models of PD. [23] To determine the mechanism(s) by which exercise mitigates the signs of PD in humans, we must first establish an appropriate dosage of exercise with beneficial effects confirmed in a Phase 3 clinical trial.

In 2018, we published findings from a multicenter Phase 2 clinical trial, the Study in Parkinson Disease of Exercise (SPARX), using a futility design, in which we studied the feasibility of having participants with PD perform moderate (60–65% maximum heart rate (HRmax)) and high-intensity endurance exercise (80–85% HRmax) 4 days per week for 6 months [24, 25]. Participants had not yet started dopaminergic medication, eliminating the potential confound of medication effects over time. We demonstrated that (1) participants could exercise at 60–65% or 80–85% of HRmax for at least 6 months, (2) they exercised for at least 3 days per week, (3) adverse events were low and consistent with those expected for endurance exercise, and (4) exercising at 80–85% HRmax slowed PD progression enough compared to usual care to warrant further investigation for efficacy at that intensity. Progression was not slowed for participants exercising at 60–65% HRmax to warrant further investigation for efficacy. These 4 findings were deemed a priori to be the necessary results to proceed to a Phase 3 efficacy trial. As such, we are now conducting a Phase 3 efficacy trial to test high-intensity treadmill endurance exercise for slowing the progression of the signs of PD.

## Objectives {7}

This Phase 3 clinical trial will test whether the progression of the motor signs of PD, as measured by the Movement Disorders Society Unified Parkinson’s Disease Rating Scale (MDS-UPDRS) motor score (part III), is attenuated at 12 months in people with PD who have not yet started dopaminergic medication when they perform high-intensity endurance treadmill exercise compared to those who perform moderate-intensity treadmill exercise. Secondary objectives are to test (1) whether there is a reduction in the percent decline of the striatal dopamine transporter binding at 12 months, (2) whether the progression of motor symptoms is attenuated when they continue to perform endurance treadmill exercise training at 18 months, and (3) the effects of endurance exercise training on ambulatory mobility, daily walking activity, cardiorespiratory fitness, quality of life, cognition, time to initiate dopaminergic therapy and dose of dopaminergic medication, blood-derived biomarkers of inflammation, and neurotrophic factors at 12 and 18 months. Tertiary objectives are to compare specific characteristics of ambulation at 12 and 18 months between the high-intensity and moderate-intensity exercise groups. An exploratory objective is to test whether the progression of the signs of PD is also attenuated at 24 months, 6 months after supervised exercise is discontinued.

## Trial design {8}

**Fig. 1:**
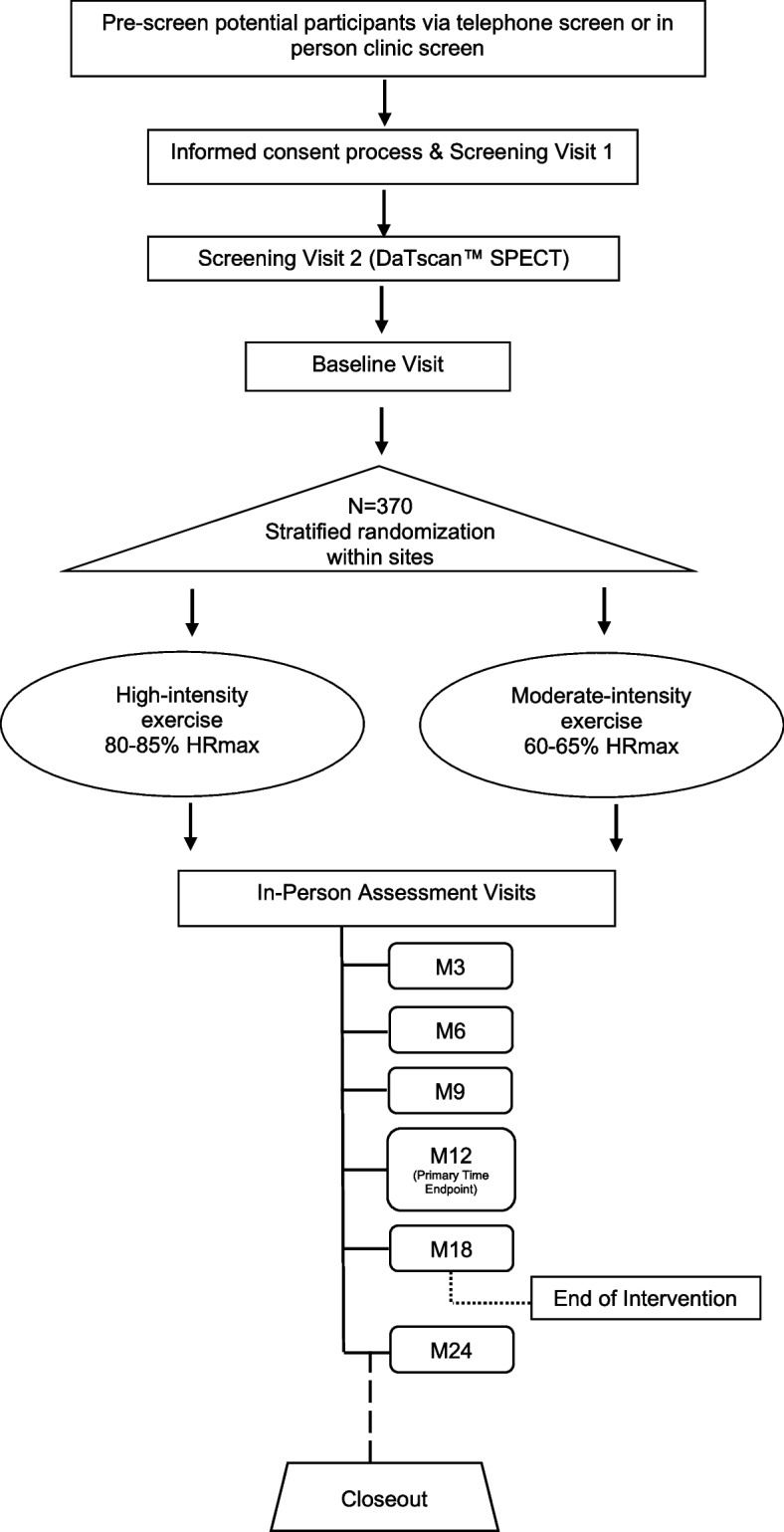
Design of the Study in Parkinson Disease of Exercise (SPARX3) Trial. The moderate-intensity exercise group is assigned to exercise 4 days a week at 60–65% HRmax, and the high-intensity exercise is assigned to exercise 4 days a week at 80–85% HRmax. The duration of the intervention is 18 months. Months 19 to 24 are observational

SPARX3 is a Phase 3, multisite, randomized, two-arm (1:1 allocation), parallel group, evaluator-blinded, clinical trial to test the superiority hypothesis that high-intensity, endurance treadmill exercise slows the progression of the signs of PD compared to moderate-intensity endurance treadmill exercise. Assessments occur at baseline and at 3, 6, 9, 12, 18, and 24 months (Fig. 1).

## Methods: participants, interventions, and outcomes

### Study setting {9}

This study is being conducted at 26 clinical sites across all geographic regions of the USA and 2 clinical sites in Canada (28 sites total). Nearly all clinical sites are affiliated with academic medical centers, having neurology practices specializing in movement disorders, as well as exercise physiology laboratories and/or physical therapy clinics. The list of study sites can be obtained from ClinicalTrials.gov, and on the study web page https://www.sparx3pd.com. Screening and assessment visits are occurring in both neurology and exercise laboratory settings.

### Eligibility criteria {10}

Our study population is defined as persons 40–80 years of age with a recent (less than 3 years) diagnosis of PD who are untreated with PD medications at baseline and are not expected to require PD pharmacologic treatment within 6 months of starting the study [24]. Potential participants undergo a four-step screening process, comprised of a phone or clinic interview, an in-person medical screening visit, confirmation of idiopathic PD with imaging, and an in-person baseline assessment to ensure they meet eligibility criteria (Table 1).

**Table 1:** Inclusion and exclusion criteria for Study in Parkinson Disease of Exercise (SPARX3) Trial

Inclusion criteria	
1. A diagnosis of idiopathic PD based on the modified *UK PD brain bank criteria [26, 27, 28] and which are consistent with recent criteria proposed for clinically established early established Parkinson’s disease that no longer exclude individuals with a family history of Parkinson’s disease [29]. 2. Hoehn and Yahr stage: less than 3 3. Disease duration: less than 3 years since disease diagnosis 4. Age: 40–80 years 5. Positive DaTscan™ SPECT by qualitative visual assessment from the Institute of Neurodegenerative Disorders. i. For women: If not surgically sterile or postmenopausal, a negative pregnancy test will be required prior to receiving the DaTscan™ SPECT.	
Exclusion criteria	
1. Currently being treated with PD medications such as levodopa or dopamine receptor agonists, MAO-B inhibitors, amantadine, or anticholinergics. 2. Expected to require treatment with medication for PD in the first 6 months of the study. 3. Use of any PD medication 60 days prior to the baseline visit including but not limited to levodopa, direct dopamine agonists, amantadine, Rasagiline (Azilect), Selegiline (Eldepryl), Artane (trihexyphenidyl), Mucuna. 4. Duration of previous use of medications for PD exceeds 60 days. 5. Use of neuroleptics/dopamine receptor blockers for more than 30 days in the year prior to baseline visit, or any use within 30 days of baseline visit. 6. Presence of known cardiovascular, metabolic, or renal disease or individuals with major signs or symptoms suggestive of cardiovascular, metabolic, or renal disease without medical clearance to participate in the exercise program. 7. Uncontrolled hypertension (resting blood pressure is greater than 150/90 mmHg). 8. Individuals with orthostatic hypotension and standing systolic BP below 100 will be excluded. Orthostatic hypotension (OH) is a reduction of systolic blood pressure of at least 20 mm Hg or diastolic blood pressure of at least 10 mm Hg within 3 min of standing. 9. Hypo- or hyperthyroidism (TSH is less than 0.5 or is greater than 5.0 mU/L), abnormal liver function (AST or ALT more than 2 times the upper limit of normal), abnormal renal function (creatinine clearance calculated by the Cockcroft-Gault equation is less than 50mL/min, or estimated glomerular filtration rate using the MDRD4 equation or the CKD-EPI equation is less than 45 mL/min/1.73 m^2^). 10. Complete Blood Count (CBC) out of range and physician’s judgment that abnormal value is clinically significant. 11. Recent use of psychotropic medications (e.g., anxiolytics, hypnotics, benzodiazepines, antidepressants) where dosage has not been stable for 28 days prior to screening. 12. Serious illness (requiring systemic treatment and/or hospitalization) within the last 4 weeks. 13. Any other clinically significant medical condition, psychiatric condition, drug or alcohol abuse, assessment or laboratory abnormality that would, in the judgment of the investigator, interfere with the subject’s ability to participate in the study.	1. Montreal Cognitive Assessment (MoCA) score of less than 24. 2. Beck Depression Inventory II (BDI) score is greater than 28, indicating severe depression that precludes ability to exercise. Any subject with such a score will be referred to a Primary Care Physician (PCP) or physician for further evaluation and management of depression. Individuals with a BDI-II score of 17-28 will be excluded if any of the following conditions are met: (1) individual is suicidal, (2) needs depression treatment modification currently or (3) depressive symptoms are likely to interfere with adherence to study protocol. Any subject with such a score will be referred to a PCP or physician for further evaluation and management of depression. 3. Individuals who have been exercising at greater than moderate intensity for 120 min or more per week consistently over the last 6 months will be excluded. Greater than moderate intensity is defined as a range greater than 60–65% HRmax. These individuals are excluded since their exercise activities are greater than the activities they would experience if they were assigned to the 60–65% treatment group. As such, they would be expected to lose fitness. 4. Use of the following within 90 days prior to the DAT neuroimaging screening evaluation: bupropion, modafinil, armodafinil, metoclopramide, alpha-methyldopa, methylphenidate, reserpine, any amphetamine or amphetamine derivative. These can compromise DaTscan™ SPECT. 5. Known allergy to iodinated products. 6. Known hypersensitivity to DaTscan™ SPECT (either to the active substance of ^123^I-ioflupane or any of the excipients). 7. (For women only) Actively breastfeeding an infant, and/or pregnant, or plan to become pregnant in the next 12 months. 8. Other disorders, injuries, diseases, or conditions that might interfere with the ability to perform endurance exercises (e.g., history of stroke, respiratory problems, traumatic brain injury, orthopedic injury, or neuromuscular disease).

### Who will take informed consent? {26a}

Standard written informed consent is obtained at the first in-person screening visit. Those who elect to participate are given the informed consent to review. The informed consent form is explained in detail by a study site research team member, including but not limited to the study purpose, duration, procedures, risks, benefits, confidentiality, instructions on whom to contact with questions, and the voluntary nature of participation. We do not permit consent from a legally authorized representative for this study. In addition to the research participant, written informed consent must be signed by a site investigator or authorized research team member. As part of clinical monitoring, the Clinical and Data Coordinating Center (CDCC) acquires documentation of the consent process and 100% of the informed consent forms are monitored remotely.

### Additional consent provisions for collection and use of participant data and biological specimens {26b}

There are no required additional consent provisions for collection and use of participant data and biological specimens in ancillary studies. Participants are encouraged to take part in the Parkinson’s Progression Markers Initiative (PPMI). Participants are encouraged to take part in PD GENEration (https://www.parkinson.org/PDGENEration) so that the genetic data can be integrated and used with SPARX3 data. Consenting for PPMI and PD GENEration is independent of consenting for SPARX3.

## Interventions

### Explanation for the choice of comparators {6b}

Exercise regimens include four principles of dosing: (1) frequency, (2) intensity, (3) length of time of exercise session, and (4) type [30]. With respect to people with PD, an additional principle of exercise delivery is whether exercise is performed at a high experimenter-controlled cadence or not [31]. We have chosen (1) the frequency of exercise to be 4 times per week, (2) a moderate-intensity of 60–65% HRmax or high-intensity of 80–85% HRmax, (3) the exercise time to be 30 min (with an additional 5-min warm up and a 5-min cool down), and (4) the type to be endurance treadmill exercise without experimenter-controlled cadence. We are using a treadmill to ensure participants follow a regular rhythmic stimulus that is provided by the moving treadmill using procedures similar to our Phase 2 study [24]. HRmax is measured during the baseline maximal graded exercise test (GXT) and is used to determine the appropriate heart rate training zone for each participant based on the exercise group to which they are randomized. It is particularly important to accurately measure each participant’s HRmax in research studies because formulas for estimating the HRmax are no longer recommended [30], and may not be accurate for all people with PD due to the possibility of a blunted heart rate response [32].

The 60–65% HRmax was chosen as the comparator based on the SPARX Phase 2 study which showed feasibility and safety for 6 months but was deemed futile in slowing disease progression compared to usual care. We considered four other control groups including (1) an intensity lower than moderate (57.5–62.5%), (2) best medical management (usual care/no study prescribed exercise), (3) a stretching, balance, and light resistance group, and (4) a placebo drug group. We chose to use a moderate intensity of endurance exercise as a clear test of the hypothesis that the intensity of the dose of endurance exercise is important. This also narrows the focus on a potential disease-specific biological effect, as opposed to a general symptomatic benefit due to improvements in fitness and agility.

### Intervention description {11a}

The frequency, time, and type of exercise for the high-intensity group are the same as the moderate-intensity comparator group: treadmill walking 4 times per week for 30 min with an additional 5-min warm up and 5-min cool down. The targeted HR range for the high-intensity group is 80–85% HRmax. The 80–85% HRmax was originally chosen because of increases in VO_2peak_ at this exercise intensity in healthy individuals of a similar age to those who typically have PD (age range 60–71) [33]. This is close to the highest heart rate that typical adults can exercise on a continuous and sustained basis [34]. The Study in Parkinson Disease of Exercise (SPARX), a Phase 2 randomized clinical trial, extended this finding to people with PD who had not previously been medicated [24]. The high-intensity group undergoes exercise training to promote physiological adaptations using treadmill speed and/or incline.

For both exercise intervention groups, the initial training occurs at exercise laboratories where exercise interventionists instruct participants to monitor heart rate and adjust the exercise intensity to remain in the target heart rate range (i.e., by changing treadmill speed and/or incline). Qualified study personnel (exercise physiologist, physical therapist, or study coordinator) responsible for implementing the exercise intervention are required to review the SPARX3 Exercise Intervention Training Manual, attend or review a 1-h training webinar, and complete a certification exam prior to obtaining approval to work with participants. Within approximately 1 week of randomization, participants should begin exercise under supervision of the exercise interventionist. The exercise interventionist provides each participant an orientation regarding treadmill utilization, treadmill safety, and heart rate monitor training. Participants wear a heart rate monitor (Zephyr^TM^ bioharness 3.0 heart rate monitor) that captures and stores heart rate and step cadence throughout exercise bouts [35]. Over the first few weeks, the exercise interventionist helps the participant identify a combination of speed and/or incline that achieves the targeted heart rate intensity. In-person exercise supervision takes place until the participant is deemed independent, after which participants are allowed to complete the treadmill exercise sessions at a fitness facility or on their own at home. Participants are required to complete in-person exercise sessions under the supervision of the exercise interventionist periodically (approximately once per month) throughout the 18-month intervention, which can occur at the research facility, in fitness facilities or at home.

### Criteria for discontinuing or modifying allocated interventions {11b}

Participants with PD may be taking medications that lower their HRmax (such as beta blockers) or may be prescribed such medications during the study. Participants who start, stop, or change the dose of chronotropic medications during the study will have exercise intensity monitored using ratings of perceived exertion (RPE) they were using prior to the change in medication until a new maximum heart rate is assessed at the next assessment visit. It is important that participants are instructed how to appropriately calibrate their RPE response during their exercise training sessions so that their RPE is consistent should they need to exercise using RPE [36].

If a clinically significant medical finding is identified or the participant experiences adverse effects during the exercise phase of the protocol, the investigator or qualified designee will determine any changes to the continuation of exercise. Participants are free to withdraw from the intervention at any time upon request. All temporary or permanent discontinuations or modifications to the exercise intervention are documented along with the reason(s) for the changes.

### Strategies to improve adherence to interventions {11c}

The exercise coordinators view the HR monitor data via a cloud-based platform provided by the service provider (Zephyr, Medtronic, Inc.). In addition, data from each exercise session (both supervised and unsupervised) are integrated into the study database. During supervised sessions, the exercise coordinators review and discuss adherence to the intervention with the participant to provide feedback and identify and provide solutions to barriers of exercise participation. To enhance long-term adherence to exercise, we are allowing participants to exercise at a facility of their choice or at home and are paying the cost of the facility or treadmill equipment needed for in-home use, when necessary. The in-home option is particularly important in the era of COVID-19.

### Relevant concomitant care permitted or prohibited during the trial {11d}

During this study, participants are asked to refrain from enrolling in any interventional studies or other studies that could affect their MDS-UPDRS motor score (Part III) or VO_2peak_ (e.g., drug trials, exercise studies), but can continue to engage in their usual physical activities prior to enrolment in SPARX3. Participants are encouraged not to take dopaminergic medication for the duration of the study, unless medically necessary.

### Provisions for post-trial care {30}

There are no provisions for post-trial care due to the nature of the intervention. Those participants who received a treadmill for home exercise will be able to keep the treadmills for their personal use after the trial is over but will be required to report the current value of the treadmill to the Internal Revenue Service. If the high intensity is found to be efficacious, those participants exercising at moderate intensity will be able to increase their intensity using the study treadmill.

### Outcomes {12}

The outcome measures for SPARX3 are listed below as well as in Table 2 which includes the domain, measure, metric, method of aggregation, and timepoint.

**Table 2:** SPARX3 trial outcomes

	Domain	Specific measurement variable	Metric	Method of aggregation	Timepoint(s)
**Primary outcome**					
(1) MDS-UPDRS part 3	Motor sign assessment of Parkinson’s disease	MDS-UPDRS part III	Score at time point assessed	Mean	12 months
**Secondary outcomes**					
(1) DatScan (Dopaminergic activity)	Dopamine neuron function (Brain Imaging)	Striatal specific binding ratio	Value at time point assessed	Mean	12 months
(2) Six min walk	Functional capacity	Distance walked in 6 min in meters	Value at time point assessed	Mean	12 and 18 months
(3) MDS-UPDRS part 3	Motor sign assessment of Parkinson’s disease	MDS-UPDRS part III	Score at time point assessed	Mean	18 months
(4) Activity Level	Physical activity	Number of daily steps	Value at time point assessed	Mean	12 and 18 months
(5) Cognitive Function	Cognitive function	Montreal Cognitive Assessment Scale	Score at time point assessed	Mean	12 and 18 months
(6) Peak VO2	Cardiorespiratory fitness	Peak volume of oxygen consumed	Value at time point assessed	Mean	12 and 18 months
(7) The Parkinson Disease Questionnaire (PDQ-39)	Quality of life	PDQ-39	Score at time point assessed	Mean	12 and 18 months
(8) Initiation of dopaminergic therapy	Symptom progression	Time (months)	Month of drug initiation	Time to event	---
(9) C-Reactive Protein	Inflammation	CRP protein (mg/L)	Value at time point assessed	Mean	12 and 18 months
(10) Brain-derived neurotrophic factor	Neuronal function and survival	BDNF protein (ng/ml)	Value at time point assessed	Mean	12 and 18 months
**Tertiary outcomes**					
(1) Stride Length	Gait	Length of stride (meters)	Value at time point assessed	Mean	12 and 18 months
(2) Turning Velocity	Gait	Turning velocity (degrees/second)	Value at time point assessed	Mean	12 and 18 months

#### Primary outcome

The primary efficacy outcome is the MDS-UPDRS motor examination score (Part III) at 12 months. The MDS-UPDRS (Parts I–IV) is used to evaluate various aspects of PD including non-motor and motor experiences of daily living and motor complications [37]. The MDS-UPDRS Part III is a 33-item rater-assessed evaluation of motor signs with each item rated 0 to 4. The motor examination score is created by summing the ratings with higher scores indicating worse motor signs. Twelve months was selected as the primary time end point as a longer-term outcome compared to the Phase 2 trial with hypothesized trajectories of the two intervention groups based on the Phase 2 6-month changes and a sample of people with PD excluding people with scans without evidence of dopamine deficit (SWEDD) [38, 39].

#### Secondary outcomes

Secondary outcomes include brain imaging, motor sign severity (longer term), functional capacity, physical activity, cognitive function, cardiorespiratory fitness, quality of life, symptom progression, inflammation, and neuronal function and survival.

- Dopamine Neuron Function (Brain Imaging): The striatal specific binding ratio (SSBR) is quantified with dopamine transporter (DAT) single photon emission tomography (SPECT) imaging with DaTscan™ occurring at local imaging centers with central processing and review by the contract research organization Invicro at screening and 12 months. Recent publications from a multisite observational study in PD, the Parkinson’s Progression Markers Initiative (PPMI) suggest that quantified DAT binding may provide a valuable tool in assessing mechanisms from interventions designed to slow progression of the disease, especially early in the disease [40, 41, 42].
- Motor Sign Severity: The MDS-UPDRS motor score (Part III) at 18 months.
- Functional Capacity: The total distance walked in 6 min is used as a measure of ambulatory mobility [43]. Using standardized courses and instructions, participants are instructed to walk as far as possible for 6 min and the distance walked is measured in meters at 12 and 18 months [44, 45, 46].
- Physical activity: Daily walking activity is measured by the number of daily steps obtained from thigh mounted activity monitors (activPAL™). Participants wear an activity monitor for 1 week every 3 months to assess average daily step count. Time points of interest are at 12 and 18 months.
- Cognitive function: The Montreal Cognitive Assessment (MoCA – version 7.1) assesses different cognitive domains of attention and concentration, executive functions, memory, language, visuo-constructional skills, conceptual thinking, calculations, and orientation. The total score at 12 and 18 months are secondary outcomes where higher scores indicate better cognitive function.
- Cardiorespiratory fitness: VO_2peak_ (ml/kg/min) is the gold standard of cardiorespiratory fitness and is considered a vital sign linked to all-cause mortality [47]. VO_2peak_ is obtained using a maximal graded exercise test (GXT). The values at 12 and 18 months are secondary outcomes with higher values indicating better fitness.
- Quality of life: Quality of life will be measured with self-reported Parkinson’s Disease Questionnaire-39 (PDQ-39) at 12 and 18 months. The PDQ-39 has 8 subscales representing mobility, activities of daily living, emotional well-being, stigma, social support, cognition, communication, and bodily discomfort [48].
- Symptom progression: The time to initiate dopaminergic therapy is defined as the time from randomization to the time of initiation of dopaminergic therapy in months. The doses of medications will be converted to levodopa equivalent doses (LED) for purposes of quantifying the amount for analysis.
- Inflammation and neuronal function and survival: C-reactive protein (CRP, mg/L) and brain-derived neurotrophic factor (BDNF, ng/ml) will be assessed in peripheral blood. CRP is elevated in PD [49], and BDNF is reduced and associated with cognitive impairments [50]. The effects of endurance exercise have yet to be studied on CRP in PD, but exercise reduced CRP in older adults [51]. A high-intensity bootcamp for people with PD demonstrated clinical improvement that was associated with increased BDNF and an anti-inflammatory response [19]. BDNF has been shown to respond to exercise in PD [52].

#### Tertiary outcomes

Gait characteristics of stride length and turning velocity will be measured at 12 and 18 months. Participants wear 5 Opal inertial measurement units (APDM Inc, Portland, OR) (feet, wrists, and lumbar area) for the 6-min walk from which we derive 2 tertiary measures related to gait: stride length and turning velocity. Both are potentially sensitive measures of the beneficial effects of exercise and gait impairment in recently diagnosed people with PD and as such may differentiate treatment arms [53, 54].

#### Additional measures

SPARX3 provides an outstanding opportunity to investigate several other outcomes and measures that may respond to exercise, inform exercise dose response, diagnosis, biological change, and responsiveness to exercise with respect to genetic profiles.

MDS-UPDRS Part I and Part II are measures of non-motor experiences of daily living and motor experiences of daily living, respectively, with higher scores indicating more disease burden. The Schwab and England Activities of Daily Living scale is a single rating of a person’s ability to complete activities of daily living. The Quality of Life in Neurological Disorders (Neuro-QOL) is being collected so that SPARX3 can contribute to describing and comparing the quality of life among persons with Parkinson’s disease to those with other neurological conditions [55]. We assess both patient and clinician global impression of change (P-GIC, C-GIC) as well as whether PD pharmacologic therapy is warranted. The P-GIC and C-GIC both range from 1 (Very much improved) to 7 (Very much worse). As explanatory variables, we document both treadmill incline and speed since these may inform adverse events. We measure the cadence at which people walk on the treadmill using the Zephyr monitor since evidence suggests that the cadence with which a person cycles on a stationary bike may be a key variable in reducing the signs of PD. [31, 56] We collect a comprehensive set of measures to allow us to document safety and adherence, as well as information on all participant medications, paying particular attention to chronotropic medications which may affect how participants respond to endurance exercise due to reduction in HRmax. The Zephyr^TM^ bioharness provides a wide variety of measures related to cardio autonomic dysfunction which may be impaired in some people with PD. [57] The extent to which autonomic dysfunction modulates how a person responds to exercise remains unknown.

For blood biomarkers, we have aligned our protocols closely with the Parkinson’s Progression Markers Initiative (PPMI) and matched healthy control groups so that our data can be compared with and complement each other. All blood samples including whole blood, plasma, serum, and buffy coat for DNA are sent to BioSpecimen Exchange for Neurological Disorders (BioSEND) [58] and are stored using state-of-the-art techniques (more details on collection and storage in designated section “Plans for collection and storage of biological specimens for genetic, biochemical or molecular analysis in the future {33}”). In plasma and serum, we plan to measure markers that may help us with diagnosis, prognostication of progression, monitoring disease progression, predicting response to exercise intervention, and assessing biological response to exercise [59].

Finally, the Exercise Confidence Beliefs & Goals questionnaire is administered at the baseline visit and will allow us to predict exercise adherence and compliance with the intervention. This is a battery of questionnaires that align with Social Cognitive Theory [60]. We are measuring exercise self-efficacy, outcome expectations, facilitators/barriers, and goal setting/planning [61, 62, 63, 64, 65]. This will also inform future exercise trials allowing for optimization of the variables for maximizing compliance and training adaptations.

### Participant timeline {13}

The complete schedule of pre-screening, enrollment, randomization, interventions, assessments, and visits for participants is provided (Table 3). Of note, the symptomatic treatment visit is an “as needed” visit to obtain an assessment of the MDS-UPDRS just prior to initiating dopaminergic therapy, should the participant plan to start dopaminergic medication. Initiating dopaminergic is not part of the plan for all participants but this will happen for some under the medical care of their neurologist or primary care physician. If a participant initiates dopaminergic medications, all subsequent MDS-UPDRS assessments will be administered in the medication “OFF” state, with dopaminergic therapy withheld for at least 12 or 24 h depending on the medication prior to assessment.

**Table 3:** Timeline of schedule of activities for Study in Parkinson Disease of Exercise (SPARX3) trial

	Pre-Screen	Screening 1	Screening 2	Baseline	Month 1	Month 2	Month 3	Month 4	Month 5	Month 6	Month 7	Month 8	Month 9
**Activity**													
Pre-Screen (phone or clinic)	X												
Informed Consent		X											
Demographics		X											
General & PD Medical History		X											
Physical/Neurological Exam		X								X			
PD/Non-PD Medication Logs		X		X			X			X			X
Beck’s Depression Inventory (BDI-II)		X											
Montreal Cognitive Assessment		X								X			
Blood Draw for Exercise Clearance		X											
DaTscan™ SPECT			X										
DaTscan™ Safety Follow-Up			X										
Inclusion/Exclusion Review		X	X	X									
MDS-UPDRS (I, II, III, IV) with H&Y				X			X			X			X
Schwab and England				X			X			X			X
6-Min Walk Test with OPALS				X						X			
Activity Monitor (daily steps)			X				X			X			X
Neuro-QOL				X						X			
PDQ-39				X						X			
VO_2_peak				X						X			
Blood Draw for Biomarkers				X						X			
Health Status Update				X	X	X	X	X	X	X	X	X	X
Modified Health Status Update													
Exercise Confidence Beliefs & Goals				X									
Randomization				X									
PD Therapy Warranted										X			
Patient Global Impression of Change										X			
Clinician Global Impression of Change										X			
Intervention: 4×/week treadmill exercise					X	X	X	X	X	X	X	X	X
Exercise Supervision & HR Data Verification					X	X	X	X	X	X	X	X	X
Treadmill Speed & Incline							X			X			X
Intervention Initiation					X								
Study Discontinuation/Completion													
Patient Stipend		X	X	X			X			X			X

	Month 10	Month 11	Month 12	Month 13	Month 14	Month 15	Month 16	Month 17	Month 18	Months 19-23	Month 24	Symptomatic T_x_
**Activity**												
Pre-Screen (phone or clinic)												
Informed Consent												
Demographics												
General & PD Medical History												
Physical/Neurological Exam			X						X			
PD/Non-PD Medication Logs			X						X			X
Beck’s Depression Inventory (BDI-II)												
Montreal Cognitive Assessment			X						X			
Blood Draw for Exercise Clearance												
DaTscan™ SPECT			X									
DaTscan™ Safety Follow-Up			X									
Inclusion/Exclusion Review												
MDS-UPDRS (I, II, III, IV) with H&Y			X						X		X	X
Schwab and England			X						X		X	X
6-Min Walk Test with OPALS			X						X			
Activity Monitor (daily steps)			X			X			X			
Neuro-QOL			X						X		X	
PDQ-39			X						X		X	
VO_2_peak			X						X			
Blood Draw for Biomarkers			X						X		X	
Health Status Update	X	X	X	X	X	X	X	X	X			
Modified Health Status Update										X	X	
Exercise Confidence Beliefs & Goals												
Randomization												
PD Therapy Warranted			X						X			X
Patient Global Impression of Change			X						X			
Clinician Global Impression of Change			X						X			
Intervention: 4×/week treadmill exercise	X	X	X	X	X	X	X	X	X			
Exercise Supervision & HR Data Verification	X	X	X	X	X	X	X	X	X			
Treadmill Speed & Incline			X						X			
Intervention Initiation												
Study Discontinuation/Completion											X	
Patient Stipend			X						X		X	X

### Sample size {14}

The primary objective of this Phase 3 randomized clinical trial is to test if high-intensity endurance exercise reduces the progression of the signs of PD at 12 months compared to moderate-intensity endurance exercise as measured by the MDS-UPDRS motor score (Part III). Based on SPARX Phase 2 data, we hypothesize the high-intensity group will have little to no worsening at 12 months. In addition, we predict that the moderate-intensity group will worsen by at least 3.5 points at 12 months [24]. We would expect the moderate-intensity group to progress no more than the PPMI cohort which showed 12-month changes ranging from 4.2 in untreated and treated patients in OFF state [40] to 6.3 in untreated patients [66]. If we conservatively assume a standard deviation of 8.2 (high-intensity exercise) [24], with a minimum sample size of *N*=240, we will have 91% power to detect a difference of 3.5, which is in the range of the minimal clinically important differences for change on the MDS-UPDRS motor score (Part III) (*α*=0.05) [67]. If we adjust for 10% lower-adherence in the high-intensity group based on the SPARX Phase 2 [240/(1 − 0.1)^2=296] and inflate for 20% attrition at 12 months (296/0.8), we will need to randomize *N*=370 participants. The difference of 3.5 is entirely consistent with two recent studies published since SPARX3 was funded in 2019 [68, 69]. Van der Kolk and colleagues showed a between-group difference of 4.2 points on the MDS-UPDRS motor score (Part III) at 6 months when comparing high-intensity endurance exercise with stretching (*n*=65 per group) [68]. Similarly, Mak and colleagues showed a difference of 4.6 points (*n*=35 per group) for a brisk walking and balance intervention [69]. The smaller sample size of these two studies compared to SPARX3 is probably due to the fact that the control group used by Van der Kolk and colleagues was assigned a less vigorous intervention than our control group and the treatment intervention used by Mak and colleagues included two interventions [68, 69]. For our secondary outcomes (analyzed as continuous variables), we will have 80% power to detect a small effect size (0.36, approximately one-third standard deviation) with at least *n*=120 per group at 12 months.

For time to dopaminergic initiation, 27% of participants in the SPARX Phase 2 initiated PD medications prior to 12 months of follow-up which is low compared to NINDS Exploratory Trials in PD (NET PD) (48%) and PPMI (59%) studies [66, 70]. Although we have assumed 20% attrition at 12 months, we expect to have at least *N*=260 with some follow-up information (*n*=130 participants per group) during the 12-month follow-up to conduct analyses on starting dopaminergic medication. We will have 84% power to detect an absolute 15% reduction in the proportion initiating dopaminergic therapy prior to 12 months in the high-intensity exercise group assuming a 30% dopaminergic initiation rate in the moderate-intensity group (two-side test of proportions, *α*=0.05). The power for the 15% absolute reduction is 74 and 70% if the moderate-intensity group rate is 40 and 50%, respectively. All sample size analyses were conducted using PASS version 15 (Power Analysis and Sample Size Software (2017)).

### Recruitment {15}

The Parkinson Study Group (PSG), which has conducted many key intervention studies of treatments for Parkinson’s disease since 1987 (https://www.parkinson-study-group.org/clinical-trials), has approved SPARX3 as a PSG study. Most of the sites were PSG-credentialed prior to funding of SPARX3, and all other sites were credentialed before their activation. Prior to submitting the grant application, 29 sites were very carefully vetted to maximize the probability that SPARX3 would be able to recruit the required number of participants. Should enrollment fall behind schedule, PSG has more than 120 additional credentialed North American sites that may be enlisted to help with recruitment.

A Recruitment, Retention and Diversity Core (RRDC) is responsible for training sites on recruitment strategies with a particular focus on increasing the proportion of minorities in the SPARX3 trial compared to previous trials in PD. [71, 72] Attaining an adequate representation of diverse populations ensures equity and generalizability and may allow for identification and evaluation of racial/ethnic differences in response to the intervention. The RRDC developed a series of 5 minority recruitment training modules delivered to research coordinators, study neurologists, and the site principal investigators at the beginning of site recruitment. The 5 modules focus on the importance of recruiting ethnically and racially diverse participants, identifying barriers to minority recruitment, developing process improvement plans, locating areas with potential minority participants, strategies for communicating with community physicians to address barriers to physician referrals, effective patient-focused communication practices for improved study recruitment, and implementing navigation strategies to address participant barriers. The RRDC will monitor targeted and actual enrolment using reports generated by the Data Coordinating Center and through discussions with the sites during regular check-in calls. The RRDC and the CDCC will work with sites that fall below their individual site targeted enrolment numbers to problem solve on barriers and challenges to recruitment.

## Assignment of interventions: allocation

### Sequence generation {16a}

Participants are randomized 1:1 to (1) exercise 4 times per week at 60–65% HRmax or (2) exercise 4 times per week at 80–85% HRmax. The study statistician generates the randomization list in SAS version 9.4 using permuted blocks of random block sizes stratified by site.

### Concealment mechanism {16b}

The systems analyst will load the list into the web-based data management system such that allocation will only be revealed once the participant is deemed eligible based on entered eligibility information and agrees to be randomized.

### Implementation {16c}

An unblinded study member will retrieve the allocation from the electronic data capture system (EDC) and ensure implementation of the correct exercise intensity arm.

## Assignment of interventions: blinding

### Who will be blinded {17a}

Due to the nature of the intervention, trial participants are not blinded to assigned exercise intensity. Participants are instructed not to discuss their exercise with study personnel except the exercise coordinator. Any research personnel responsible for scoring the assessments for primary or secondary outcomes are blinded to group assignment. All personnel conducting assessments are trained not to discuss any part of the exercise intervention with the participants. The project coordinator, the quality control lead, and study team members involved with the exercise implementation and supervision are aware of the exercise arm. All other investigators, including the study principal investigator (PI), site PIs, and research staff, remain blinded to intervention allocation for participants. No study-wide reports contain information about intervention arms. An independent doctoral level statistician and masters level analyst will generate closed reports for the Data Safety Monitoring Board (DSMB) stratified by coded intervention arms (A or B).

### Procedure for unblinding if needed {17b}

There is no circumstance under which unblinding would be required or permissible at the site level. Should an evaluator become unblinded to allocation, the site is responsible for substituting a blinded evaluator for the participant’s remaining evaluations and documenting the break of blinding as a protocol deviation. The quality control and monitoring team will track these events and subsequent actions throughout the implementation of the study.

## Data collection and management

### Plans for assessment and collection of outcomes {18a}

All research investigators and staff are required to undergo extensive and documented training on the study protocol. Assessors for both the MDS-UPDRS and the Montreal Cognitive Assessment undergo separate training and certifications. Standard Operating Procedures have been developed for the 6-min walk test (including use of the Opals), VO_2peak_ test, blood draws, DAT imaging, and collection of activity monitor and heart rate data. Study team members involved with the collection and/or shipment of blood undergo additional training. All imaging centers undergo a multi-step Site Qualification Process handled by Invicro. All personnel requiring access to the EDC system are trained on the forms and data entry fields. In addition, they must successfully enter test cases for pre-screening, screening, randomization, and follow-up. The MDS-UPDRS is required to be recorded on paper and entered twice into the database to ensure the accuracy of the data since the two entries are compared. All data collection forms are in the Appendices of the Manual of Operations and available in the clinical trial document management system.

### Plans to promote participant retention and complete follow-up {18b}

The attrition rate in our prior SPARX Phase 2 clinical trial was 9% at 6 months and 16% at 12 months. We have accounted for 20% attrition in our sample size analysis. We are implementing several strategies to enhance retention and minimize loss to follow-up based on our prior experience including (a) being flexible when scheduling appointments, (b) being responsive to participants’ and/or spouse/care partner questions, and (c) ensuring participants are appropriately trained to use all equipment involved so they feel confident in their ability to engage in the appropriate exercise. The exercise coordinators meet with participants throughout the duration of the study, sometimes traveling to the participant’s fitness facility or home, which should enhance adherence to the exercise program and allow development of a strong researcher-participant relationship. We allow remote supervision periodically under certain circumstances to decrease the travel burden on participants and allow for greater flexibility when participants have vacations, work conflicts, or other events that complicate adhering to the protocol. We allow participants to choose where they exercise and provide the means to do so to promote retention and long-term adherence to exercise. A research coordinator contacts participants to inquire about their current health status at least once per month. The study team calls participants before their appointments to review study visit preparation and to remind them of the appointment time. These reminder phone calls are designed to help with retention. We provide parking passes for study visits, stipends per screening, and assessment visits and provide supervised training sessions throughout the entire duration of the study. Each participant receives a personalized thank you card signed by the study team at months 6, 12, 18, and 24 in order to acknowledge the appreciation of the study team and promote retention. Regardless of a participant’s adherence to exercise, priority will be placed on obtaining MDS-UPDRS motor score (Part III) evaluations since this is the primary outcome measure for the trial. Should a participant want to discontinue from the study, every attempt will be made to obtain a final MDS-UPDRS motor score (Part III) measure prior to study discontinuation.

### Data management {19}

Details of data management procedures can be found in the protocol under Data Collection and Management Responsibilities. Briefly, the EDC is hosted by the School of Health and Rehabilitation Sciences Data Center (SHRS DC) at the University of Pittsburgh. The EDC is a secure, web-based application developed using the Microsoft Development Stack including Microsoft Windows Server for the operating system, SQL Server for a relational database system, and the C#.NET programming language and libraries to create applications. The system provides management of user access to the data, a mechanism for validated data uploads from external sources, real-time validation rules, an audit trail for tracking data entry and edits, and a mechanism for data downloads which can be imported into statistical software for analyses.

Case report forms (CRFs) serve as the basis for the structure of the EDC with the data entry screens being as visually similar to the CRFs as possible. CRFs contain data elements matched to NINDS Common Data Elements for demographics, medical history, PD medical history, concomitant medications, quality of life measures, MDS-UPDRS, and adverse events.

For questionnaires that are self-report, a “participant mode” screen has been developed for the participant to complete the questionnaire on a tablet. Data from HR monitors are sourced from the Zephyr^TM^ cloud platform. Activity monitor data collected using activPAL™ are saved by each site to the EDC and processed at the University of Colorado. Numeric results from the quantification of the DAT imaging, biomarker data from blood assays, and genetic profiles from PD Gene will be integrated with the study data captured through the EDC.

The Clinical and Data Coordinating Center (CDCC) works with each site PI to determine access to the EDC system for each site team member. The system is protected by unique login and password. Once a research staff member has gone through data entry training and testing, and site completed all requirements to initiate recruitment, a member of the CDCC will permit access to the production system. Users will only have access to participants at their respective site. A user access log will be maintained by the CDCC.

### Confidentiality {27}

Data that could be used to identify a specific study participant is held in strict confidence by the research team. No personally identifiable information from the study will be released to any unauthorized third party without prior written approval of the sponsor/funding agency. Authorized representatives of the sponsor or funding agency, or representatives of the Institutional Review Board (IRB), may inspect all documents and records required to be maintained by the investigator for the participants in this study.

Participant’s contact information is securely stored at each clinical site for internal use during the study. Only approved team members at each site and the select members of the CDCC will have access to personal information needed for tracking and informed consent. This includes the quality control monitoring team who will monitor 100% of the informed consents. At the end of the study, all records will continue to be kept securely for the length of time required by the reviewing IRB, the site IRBs and the Research Ethics Boards (REBs), Institutional policies, and sponsor/funding agency requirements.

Each study participant is assigned a Participant Identification (PID) number. The participant names and linkage to the PID are maintained by the local study teams stored in a locked file in a locked office or in encrypted and password-protected electronic documents. No personal health identifiers (except for date of birth and hospitalization dates for serious adverse advents (SAEs)) will be entered into the EDC system. Date of birth is necessary to ensure eligibility based on calculated age at the time of screening. Monitor data (HR, activity, and movement) does not include global system positioning location of the participants. Blood sample labels do not include any identifiable information.

Participants will not be identified by name in any publications of research results. All study participants will be identified by the PID on all data collection instruments, documents, and files used in the statistical analysis and manuscript preparation. The site PIs and the CDCC ensure all mechanisms used to share data include proper plans and safeguards for the protection of privacy, confidentiality, and security for data dissemination and reuse (e.g., all data will be thoroughly de-identified and will not be traceable to a specific study participant). With the permission of the participant via the informed consent, de-identified data may be shared with other researchers from the PPMI study and/or the PD GENEration study. Additional information on protection of privacy of study participants can be found in the protocol.

### Plans for collection and storage of biological specimens for genetic, biochemical, or molecular analysis in the future {33}

With the participant’s consent, blood will be collected at each of the study visits indicated in Table 3. All samples are collected using best practices and following a study manual of procedures provided by the BioSpecimen Exchange for Neurological Disorders (BioSEND; https://biosend.org/resources/sparx3.html) [58, 73]. The protocol requires 20 ml of blood to be collected in ethylenediaminetetraacetic acid (EDTA) tubes for plasma and buffy coats (for DNA) and another 20 ml of blood collected in serum collection tubes for serum. These 40 ml of blood are spun in a refrigerated centrifuge at 4° C at 1500*g* for 15 min. The plasma and serum are aliquoted into 1.5-ml cryovials. This approach does not require intense coordinator effort to create distributable aliquots (in the 200–500 ml volume) and instead a single freeze thaw at BioSEND provides distributable aliquots that can be shipped to approved researchers for plasma and serum-based biomarker assays. An additional 6 ml of blood are collected without further processing which can be made available to researchers for future blood-based assays or for extraction of high molecular weight DNA for long-read sequencing. BioSEND provides barcoded labels for all specimens to ensure that all are de-identified [58, 73]. Only the unique participant code will be used to identify the biological sample. Care has been taken to ensure the processing procedures and collection material used are consistent with the large-scale Parkinson’s Disease Biomarkers Program (PDBP) and Parkinson’s Progression Markers Initiative (PPMI). This ensures comparability across cohorts for more robust analyses. All samples collected as part of SPARX3 can be used for future biomarker and genetic research related to the causes of PD, its complications, differential diagnosis, response to exercise, and other treatment modalities. The biological samples would be provided to researchers at academic institutions, hospitals, and biotechnology/pharmaceutical companies through a formal request process.

## Statistical methods

### Statistical methods for primary and secondary outcomes {20a}

The primary outcome is the MDS-UPDRS motor score (Part III) at 12 months. Analyses will follow intention-to-treat with all participants analyzed in the group to which they were assigned regardless of adherence. We will use linear mixed models with time (baseline, 3 months, 6 months, 9 months, 12 months, and 18 months) and the group-time interaction as fixed effects while controlling for repeated measures using an unstructured correlation matrix between time points. The effect of time will be treated as categorical. No main effect for treatment is included as the baseline means are assumed to be equal due to randomization. We will also control for site since this is a stratification factor in the randomization. Site will be added as a random effect. We will use linear contrasts to test high versus moderate intensity at 6, 12 (primary), and 18 months. If a participant initiates dopaminergic medication, MDS-UPDRS scores will be censored after initiation [74, 75]. Additional details on sensitivity analyses can be found in the Statistical Analysis Plan.

Since we have a control group that is exercising at a heart rate level that elicits clear health-related benefits [30], it is possible that there will be no differences detected between the 2 groups at 6, 12, or 18 months. In light of this, we devised an a priori plan to test the within group changes over 12 months and 18 months to see if the 95% confidence intervals exclude the mean change observed in the PPMI untreated cohort, essentially using this cohort as an historical control for each intervention group [40].

Secondary outcomes to be treated as continuous measures are distance walked during the 6-min walk test, average number of daily steps, cognitive function, VO_2peak_, and the quality-of-life subscales. These measures will be analyzed using linear mixed models like the models for the primary outcome. We will use Kaplan-Meier curves and log rank tests to compare the time to initiate dopaminergic therapy between the two groups. For those who initiate dopaminergic therapy, we will compare the dose of dopaminergic medication at initiation (converted to levodopa equivalent dose) between groups using the Wilcoxon rank sum test.

The secondary outcomes of SSBR, CRP, and BDNF are assumed to come from right-skew distributions requiring transformation prior to analysis. If the measures can be transformed to approximate a normal distribution, we will use linear mixed models using all time points to test for differences between groups. If the data are highly skewed with a large proportion of lowest level of detection prohibiting normalization, we will use a generalized linear mixed model with a log link or a tobit regression model for the comparisons.

### Interim analyses {21b}

We plan to conduct sample size re-estimation when we have approximately 50% of the targeted sample with 12-month follow-up data. The sample size re-estimation will be based on the observed variance and attrition compared to the assumed variance and attrition in the original power calculation. We will use a restricted design where the final sample size is at least as large as the originally planned sample size. This approach has negligible impact on *α* with naïve test at end of study [76]. At the same time as the sample size re-estimation, we will conduct futility analysis for the primary outcome at the primary and secondary time points (12 and 18 months) to inform the decisions for sample size should the observed variance be much higher than the variance assumed for the power analysis. The futility analysis will be conducted by an independent statistician and the results will be shared with the DSMB only. The DSMB will make a recommendation to NINDS based on these two interim analyses.

### Methods for additional analyses (e.g., subgroup analyses) {20b}

We plan to explore differences in intervention effects by sex, race/ethnicity, and PD subtypes. We will analyze our data to look for sex differences in outcomes and consistency of effects of high-intensity exercise by testing the 3-way sex-intervention-time interaction at *α* = 0.10. Although we are not adequately powered to detect a moderate sex by exercise interaction, we will have 80% power to detect moderate effect sizes (0.39–0.45) in each group given the expected proportion of men and women based on SPARX Phase 2 data and our planned target enrollment 57% men, 43% women). We expect approximately 10% of our randomized sample to be non-Caucasian or of Hispanic ethnicity. Statistical power will be extremely limited for meaningful differences should they exist. However, in the linear mixed models following intention to treat, we will conduct subgroup analyses for race (Caucasian/non-Caucasian) and ethnicity (Hispanic/non-Hispanic) by first testing the 3-way interaction at *α*=0.10 and then estimating mean differences between groups and 95% confidence intervals. PD subtypes have been identified whose rates of disease progression are different; therefore, endurance exercise may interact with the potential rates of disease progression and predominant signs may respond differently to endurance exercise. Two subtypes that we will analyze are tremor dominant (TD) and postural instability and gait disorder (PIGD). PIGD have more severe disease manifestations at diagnosis and greater cognitive progression as well as more pronounced features of dopamine dysregulation syndrome than TD patients [77, 78]. This has been interpreted as an expression of greater neurodegeneration in those who manifest the PIGD subtype at disease onset, and this could influence how people respond to exercise. We will use an interaction term with PD subtype to test if this factor modifies the response to exercise.

### Methods in analysis to handle protocol non-adherence and any statistical methods to handle missing data {20c}

We anticipate no more than 20% attrition at the 12-month assessment based on the SPARX Phase 2 study and have accounted for this in sample size analyses [24]. We will compare baseline characteristics between participants with missing 6-, 12-, and 18-month assessments to those without to assess potential biases. We will try to obtain reasons for study dropout so that we can identify potential causes for missing data. The linear mixed models proposed for analysis of the primary objective assume missing at random and have been shown to perform as well as multiple imputation given the same assumption of the missing data mechanism. We will conduct several sensitivity analyses assuming non-ignorable missingness with differential imputation [79, 80, 81] and pattern mixture models [82]. We will compare the results from these analyses to our primary analyses with all observed data to assess the robustness of our findings.

### Plans to give access to the full protocol, participant-level data, and statistical code {31c}

The full protocol and Statistical Analysis Plan (SAP) are available with this publication and will be submitted to ClinicalTrials.gov with the clinical trial results. The protocol, SAP, de-identified participant-level datasets, statistical code, and data documentation will be shared with NINDS repository within 1 year of the primary publication or within 18 months of the last study visit of the last subject, whichever occurs first.

## Oversight and monitoring

### Composition of the coordinating center and trial steering committee {5d}

This clinical trial is overseen by the Clinical Coordinating and Data Center (CDCC) and 4 Cores. The CDCC is co-directed by the PI (DMC) and the lead biostatistician (CGP). The CDCC oversees the single IRB of record and is responsible for the study protocol, training, site initiation, clinical and data monitoring, and coordination of all cores and committees as well as data management and statistical analysis. The IRB of authority for all US sites is the University of Pittsburgh. Separate REBs oversee the 2 Canadian sites. The SPARX3 Cores are the Walking Activity, Heart Rate and Exercise Monitoring Core, the Biomarker Core, the Imaging Core, and the Recruitment, Retention and Diversity Core. Each core is responsible for finalizing devices and methods, developing standard operating procedures, ensuring appropriate data elements are collected, and monitoring implementation of their specific aspects of the trial.

The SPARX3 trial requires several committees to oversee training, study implementation, and safety. The Executive Steering Committee (ESC) is comprised of the PI, the lead biostatistician, the quality control lead, Core leaders, a representative of the PSG, a patient advocate, and an NINDS Clinical Program Director. The ESC has reviewed and approved the final study protocol and will review any proposed future modifications. The ESC will monitor the study progress including recruitment, retention, and site compliance with study procedures.

The Sub-Steering Committee is composed of selected members of the Executive Steering Committee and meets at least 4 times a year. The other committees for SPARX3 are the Forms Committee (determining data elements for data collection forms), Publications Committee (policies and procedures for primary and secondary papers), Exercise Committee (training, standardization, and monitoring of the exercise procedures), Quality Control and Clinical Coordination Committee (monitor study implementation from screening to randomization and follow-up including protocol deviations and data quality), and Adverse Events Adjudication Committee (internal and external review of adverse event naming and determinations for severity, relatedness and expectedness).

### Composition of the data monitoring committee, its role and reporting structure {21a}

The Data and Safety Monitoring Board (DSMB) for SPARX3 was appointed by the National Institute of Neurologic Diseases and Stroke. An NINDS Program Official from the Office of Clinical Research serves as the NINDS liaison to the Board. The DSMB members are experts in movement disorders, endurance exercise, clinical trials, and biostatistics. The Board serves as an independent body responsible for monitoring the progress of the trial and the quality of study implementation and ensuring the safety of the participants. The charter is maintained by NINDS. The Board met to approve the protocol and meets approximately every 6 months during recruitment and follow-up. Members of the DSMB make recommendations to NINDS and the Principal Investigator concerning continuation, termination, or other modifications of the trial.

An Independent Medical Safety Monitor serves as the contact person for serious adverse event reporting and independently reviews safety-related issues that arise throughout the study. The Independent Medical Safety Monitor has the authority to remove participants from the study and take any steps to protect safety and well-being of the participants. The Independent Medical Safety Monitor for this study is a Professor of Neurology with expertise in Parkinson’s disease who was appointed prior to study implementation.

### Adverse event reporting and harms {22}

Based on the SPARX Phase II trial, we expect mainly adverse events classified as musculoskeletal and connective tissue disorders such as pain in extremity, back or buttock pain, and arthralgia as we have reported on ClinicalTrials.gov (NCT01506479). We systematically are collecting all adverse events reported by the participants every month by asking about medication changes, visits to doctor or other health care professional, hospitalizations, illness or health problems without seeing a doctor, and any problems with the exercise program. We specifically ask about fall frequency in the past month. In addition to the monthly health status update, participants may report AEs at any exercise session, research visit or in any communication with site personnel. Once an adverse event is reported by a participant, the AE is recorded in the electronic data capture system along with grade, relatedness, and expectedness following recommendations of NINDS Common Data Elements. AEs are named and graded using the Common Terminology Criteria for Adverse Events (CTCAE) version 5.0. AEs that are (i) Unexpected, (ii) Related or Possibly Related to participation in the research study, and (iii) Serious or otherwise suggests that the research places the subject or others at a greater risk of harm than was previously known or recognized, are considered reportable events. The CDCC adheres to the central IRB reporting timelines for reportable events (less than 24 h of learning of the event if fatal or life threatening; less than 10 working days of learning of the event for all other events that are not fatal and not life threatening; less than 10 working days of the investigator becoming aware of an unanticipated problem involving risk that are possibly or definitely related to the research and incidents of noncompliance that involve risk).

An Adverse Events Adjudication Committee provides an independent review of all adverse events that occur during the conduct of the trial. The committee adjudicates the adverse event name, level of severity, relatedness, and expectedness reported by the local study team resulting in consistent classification of adverse events within sites and across sites. The purpose of this external Adverse Events Adjudication Committee is to mitigate potential investigator bias and facilitate an accurate safety profile of the study.

The Independent Medical Safety Monitor will be notified of each serious adverse event with details for review to ensure appropriate clinical care and to quickly identify any potential trends. Any concerns with the SAE or SAE reporting will be relayed to the CDCC for resolution. In addition, all other individuals or entities who have oversight of the study receive an immediate notification when a SAE is submitted in the EDC.

For the primary SPARX3 publication, we will report at a minimum the number of persons with adverse events related to exercise (all and severity greater than mild), events >10% in a single group, events by organ system >10% in a single group, and number with any serious adverse events and by organ system. For ClinicalTrials.gov, all adverse events and serious adverse events will be reported by term and organ system.

### Frequency and plans for auditing trial conduct {23}

Clinical site monitoring is conducted to ensure that the rights and well-being of trial participants are protected, that the reported trial data are accurate, complete, and verifiable, and that the conduct of the trial complies with the currently approved protocol, with International Council on Harmonization Good Clinical Practice, and with applicable regulatory requirements. Monitoring of the sites is the responsibility of the PI, the Project Coordinator, and the Quality Control Lead. Monitoring is intended to take place both on-site and remotely. There are 4 types of monitoring visits for this study: Site Initiation Visits (1 visit split into 2 parts prior to site activation); Interim Monitoring Visits (annually); For-Cause Visits (as needed); and Close Out Visits (close of study). At a minimum, the participant data monitored include consent documents, SAEs, AEs, protocol deviations, and a sample of complete study files. Reports of all monitoring visits include notes of the discussions, resolution of any issues, and action items and their completion dates. These reports are distributed to site PIs and their study team and uploaded in the e-Regulatory Binder system. Due to COVID-19, all site visits have been conducted remotely.

### Plans for communicating important protocol amendments to relevant parties (e.g., trial participants, ethical committees) {25}

The study-wide communication for important protocol amendments will be the responsibility of the PI. Information will be distributed by the Project Coordinator, Regulatory Specialist, or Research Assistant on behalf of the study PI.

## Dissemination plans {31a}

The investigators are responsible for publicly disseminating results, study materials, and procedure manuals. As such, this trial is registered at ClinicalTrials.gov (NCT04284436), and results information from this trial will be submitted to ClinicalTrials.gov no later than 1 year after the study’s primary completion date. In addition, results will be disseminated through presentations and publications in peer-reviewed journals and by means of the web page: https://www.sparx3pd.com. SPARX3 has an extensive publication policy which outlines guidelines for authorship and appropriate attribution of credit to the “The SPARX3-PSG Investigators”. No professional writers will be used for SPARX3 publications. De-identified data will be submitted to the NINDS Clinical Trials repository within 1 year after publication of primary results or within 18 months of the last study visit of the last subject, whichever occurs first.

## Discussion

Phase 3 clinical trials are the benchmark for establishing treatment efficacy. In the area of PD, there have been several Phase 3 clinical trials for delaying disease progression and to date none have been successful [75, 83, 84, 85]. As such, we took great care in the design of this Phase 3 clinical trial both to maximize the probability of determining if endurance exercise should serve as first-line treatment for this population to slow disease progression, and to collect other important data to better inform the benefits of exercise. The SPARX Phase 2 clinical trial was designed to be 6 months in duration [24]. With considerable support and guidance from NINDS, the duration of the intervention of SPARX3 was tripled to 18 months. This has two effects. The first is to allow a longer time for the benefits of exercise to accrue. The second is to allow for an extended analysis of disease progression. Given that the SPARX Phase 2 clinical trial showed a difference of 3.9 points on the MDS-UPDRS motor score (Part III), that the study by van der Kolk and colleagues showed a difference of 4.2 points, and that both studies exceeded the minimally clinically important difference, our biggest concern is that increasing from 3 sites in the SPARX Phase 2 clinical trial and 1 site in the van der Kolk study will increase the variability of the MDS-UPDRS motor score (Part III). We have taken 4 approaches to mitigate this increase. First, we used the highest group specific standard deviation from SPARX Phase 2 for our sample size analysis. Second, we have required all assessors to be certified and have mandated that the same assessor is used across all 5 time points unless this is impossible. Third, we are using DaTscan™ SPECT to maximize the probability that the participants in SPARX3 have PD. It has been suggested that the rate of worsening of participants without evidence of DAT deficit (SWEDD) is less and excluding these individuals will reduce variability [86]. Fourth, as we outline in section {21B}, we will conduct an interim sample size re-estimate to make sure we are adequately powered based on the actual variability of the assessments from all sites.

### Selection of outcome measures

We chose the MDS-UPDRS motor score (Part III) to be our primary outcome since it has been more frequently used in Phase 3 clinical trials and cohort studies of disease progression in PD to date. It is not without its limitations. First, it is inherently subjective due to being rater-completed, albeit raters must undergo certification in SPARX3. Second, factors such as anxiety can influence how participants present during the examination. Third, although we closely monitor and instruct sites to use the same assessor across each timepoint, we cannot prevent staff turnover and availability for a 2-year follow-up. Fourth, it does not provide a biological assay of nervous system change. The MDS-UPDRS motor score (part III) is susceptible to the effects of dopaminergic medication. We will attempt to mitigate this by only recruiting individuals who are expected to not require dopaminergic medications for at least 6 months from enrollment, and for those who initiate medication, by assessing this score in the medication “OFF” state. We will compare dopaminergic medication doses in each study arm for those who initiate medication.

We have also used a variety of secondary and tertiary measures that may turn out to be more sensitive than the MDS-UPDRS motor score (Part III) and may provide a potential explanation of exercise-induced changes in the basal ganglia. The first is DAT binding, which has recently been shown to be quite sensitive to 12 months changes in striatal specific binding ratios (SSBRs) [87]. If there is a dose response to endurance exercise, it is possible that the typical rate of decline of about 10% of the SSBR over 12 months could be reduced. If this proves to be true, it will provide evidence that endurance exercise can attenuate the decline in dopamine binding rate in the caudate and putamen, which in turn could signify an effect of exercise on the integrity of the nigrostriatal pathway. Second, the distance walked in the 6-min walk test is sensitive to change with respect to endurance exercise [69, 88, 89]. Third, we have added two tertiary measures of gait, turning velocity and stride length, both of which are sensitive to the effects of exercise and disease progression [53, 54].

We will also be able to derive additional measures from the blood samples to either enrich the sample by excluding participants who do not have PD or to provide mechanistic insight into how exercise might work. For example, neurofilament light chain can help distinguish the atypical parkinsonisms such as multiple system atrophy and progressive supranuclear palsy from PD [90–92] whereas DAT SPECT cannot. Neurofilament light chain may also provide prognostic value by stratifying SPARX3 subjects for their cognitive and motor progression with exercise [90, 93, 94, 95]. Biomarkers responding to endurance exercise in healthy subjects will be tested to determine if their response is similar or different in PD participants and if their response correlates with their clinical outcomes from exercise. These include the secondary outcome CRP as well as interleukin-6 and other cytokines that relate to inflammation and also may relate to tremor [96]. We are assaying klotho which is an aging regulator that when overexpressed, extends life in model organisms and augments cognition [97, 98, 99]. While klotho levels decrease with aging and PD [100], physical exercise robustly increases klotho levels in healthy adults [101, 102]. Similarly, recent data show glycosylphosphatidylinositol-specific phospholipase D1 (GPLD1) is higher in older people than those who are more active (takes greater than 7100 steps daily) than in those who are less active (takes less than 7100 steps daily). Recent animal studies show GLPD1 [103] and clusterin [104] as candidate mediators of exercise benefits for the brain and would be measured as biomarkers to assess biological responses. Horowitz et al. showed that elevation of GPLD1 level by transfusing plasma from exercised mice transferred the benefit to sedentary aged mice [103]. There have also been several promising advances in exerkines and tissue-brain crosstalk [105]. Whether exercise increases these biomarkers in PD and protects against motor or non-motor signs of progression is not yet known. During the implementation of our trial over the next several years, SPARX3 is seeking additional candidate biomarkers as science advances knowledge of biological effects of exercise [106] and our understanding of how to prognosticate and monitor PD progression.

To date, there is very limited evidence to inform understanding of the extent to which genes may influence how people with Parkinson’s disease respond to exercise. A recent study utilizing data from PPMI demonstrated that increased physical activity attenuated *APOE* E4-related vulnerability to cognitive decline in individuals with PD. [107] This suggests that the benefits of physical activity may be modulated by genetic background. People who enroll in SPARX3 are encouraged to enroll in PD GENE, and these data will be available to the investigators of SPARX3 for analysis. Any analyses will be exploratory and designed to inform future studies. The genes being assessed are *LRRK2, GBA, SNCA, PRKN, PARK 7, PINK1,* and *VPS35*. The potential importance of this line of research can be understood from a recent study in PINK1-deficient *Drosophila melanogaster* which showed that exercise caused the organism’s proteomic profile to return towards wild-type levels [108]. Future DNA analysis beyond these known PD genes in the future may help us to discern the effects of genetic risk factors for progression and response to exercise as well as potentially provide subgroup analyses based on the genetic profiles.

### Participant recruitment

The most difficult aspect of clinical trials is elegantly captured by Lasagna’s Law [109]. As quoted by Feinstein, the law is as follows: “the number of patients who are actually available for a trial is about 1/10 to 1/3 of what was originally estimated” [110]. In studies of exercise and in studies of PD, there are several good reasons for this precipitous decline in the actual number of the available participants upon initiation of the clinical trial. First and foremost, SPARX3 competes with many other studies of people with PD who have not yet taken medication. The approach taken by many sites is to present all available research opportunities and let the individual choose which study or studies they prefer. The fact that sites are recruiting for many studies can impact the number of available participants, especially when these are interventional studies such as drug studies since participation in such studies is an exclusionary criterion for our study. Second, sites can be overly optimistic in their projections. Two of the three sites in SPARX Phase 2 over-estimated their anticipated recruitment numbers [24]. Prior to submitting the grant for SPARX3, all sites were formally surveyed to determine their actual ability to recruit participants for the study. Sites were asked to confirm anticipated recruitment numbers prior to study start up and final recruitment numbers were again discussed at each individual site initiation visit, as well as rate of recruitment. Third, there is certainly burden on participants when taking part in an exercise study that lasts 2 years, requires multiple assessment visits, and requires interacting with technology to measure heart rate during every exercise session. The study design we proposed was originally for 1 year, which is consistent with the participant commitment required for our Phase 2 study. The design we are implementing is twice as long requiring twice the participant commitment. Fourth, taking part in the intervention can be complicated depending on how close the participant lives either to the study site or to an exercise facility. To make taking part in the exercise intervention more feasible for all individuals, each site has the option of making treadmills available for in-home use.

Another reason why recruitment may be a challenge in SPARX3 is that, just as we did in SPARX2, SPARX3 made the decision to study participants prior to them starting medication for their PD symptoms. We made this decision primarily for 3 reasons: (1) to be able to study the effects of exercise training independent of any medication effects since medication has a clear symptomatic effect that can compromise interpretation of any results, (2) the earlier a person starts exercising, the longer the time over which exercise can potentially work to delay the progression of the disease, and (3) allows addressing the question of whether exercise can delay the time at which medication is taken. We recognize that many participants may have started medication by 12 months—the time of the primary endpoint. Nevertheless, we expect to have enough people to analyze. Our goal is to reduce this to the minimum, consistent with best research practices.

Another critical and complex clinical trial decision is determining the inclusion and exclusion criteria [111]. Ideally, the inclusion and exclusion criterion should match the population of interest being studied. However, the scientific review process is very rigorous and weighs threats to internal validity as highly, if not higher, than threats to external validity. In the original design of SPARX3, we took a very conservative approach with our exclusion criteria since we do not want our findings to be compromised by incorrect diagnosis (use of DAT imaging), medication regime, safety issues, impaired cognition, depression, comorbidities, etc. We chose to use DAT SPECT to enrich the sample with people accurately diagnosed with PD. We did not use DAT SPECT in SPARX, and to the best of our knowledge, no exercise study to date has previously used DAT SPECT for exercise studies [24]. We expect 15% of our sample to receive a negative DAT SPECT scan and thus be ineligible to participate in the study. All DAT SPECT scans conducted in SPARX3 follow strict quality assurance guidelines since the scans serve not only as a screening mechanism but also as a secondary outcome measure. DAT SPECT scans add an additional layer of complexity to exercise studies because many sites are not familiar with the procedures involved which are organizationally complex, time sensitive, and dependent on delivery of the radioactive tracer the morning of the scan.

After monitoring our screen failures very closely for the first 6 months of the study, we made 5 modifications to our exclusion criteria. First, we removed the restriction originally imposed by the central IRB to delay recruitment for anyone who had a DAT SPECT conducted in the prior 6 months. There was no safety reason behind this restriction and so we requested that it be removed since a high percentage of our participant referrals had recently had DAT SPECT imaging. Previously acquired clinical scans cannot be used in lieu of a SPARX3 scan. Second, we decreased our MoCA cutoff from 26 to 24. Third, we increased our BDI cutoff from 16 to 28, while excluding those with scores between 17 and 28 if any of the following conditions are met: (1) individual is suicidal, (2) needs depression treatment modification currently, or (3) depressive symptoms likely to interfere with adherence to study protocol. Fourth, we increased the time that participants could have taken PD medication from 30 to 60 days. Fifth, we modified our exercise criteria to exclude individuals consistently participating in 120 min or more of *greater than* moderate-intensity exercise per week over the last 6 months rather than excluding individuals exercising at moderate intensity. More and more people with PD are now exercising, and our original guidelines were too restrictive. The key criterion is that we cannot recruit people who are exercising above 60–65% HRmax since their activities are greater than the activities they would experience if they were assigned to the 60–65% HR max treatment group. As such, they would be expected to lose fitness.

To maximize the probability that we will recruit 370 participants, SPARX3 has worked very closely with the Parkinson Study Group (PSG). If recruitment accrual falls below expected rates, additional SPARX3 sites can be added. In addition, we have a Recruitment, Retention and Diversity Core who have developed 5 interactive modules to help sites recruit participants and focus on diversity [112, 113]. Historically, clinical trials in PD have had dismally low levels of minority enrollment [71, 114]. One of the SPARX3 sites is Morehouse School of Medicine which serves a predominantly Black population.

### Assuring fidelity of exercise dose prescription

Multisite studies of endurance exercise have one major logistical issue that must be addressed to ensure intervention fidelity: implementation of the VO_2peak_ test. There are many ways to establish the dose of endurance exercise and the gold standard is using a heart rate range based on the results of a laboratory based VO_2peak_ test. This test serves 2 purposes. The first purpose is to get an accurate measure of VO_2peak_ which is both a secondary outcome measure and is considered by many to be to be an intermediate outcome measure since it demonstrates the dose of treatment has had a differential effect. This differential treatment effect may be related to changes in striatal activation since Saceheli and colleagues showed endurance exercise increases both VO2_peak_ and striatal dopamine release [115]. The second purpose is to get an accurate measure of a person’s HRmax that is used to inform the heart rate prescription for the exercise. We adjust the exercise prescription if a participant’s maximal heart rate at any subsequent VO_2peak_ test assessment exceeds the previously recorded maximal heart rate by 5 beats per minute or more, which could occur if a submaximal effort was not given at a previous time point. As such, this test must be performed consistently within and across all sites. We are monitoring closely the respiratory exchange ratio (RER) within and across all sites to ensure that sites are reporting values consistent with those observed in a true peak test since we have evidence that there were site differences in RER for SPARX.

### Role of central IRB

Large multisite studies in the USA are confronted with a major logistical issue: central IRB, which is a mandated requirement. Although many local IRBs rely on the central IRB and process their paperwork in a timely fashion with only essential site-specific operational needs for change, some local IRBs request to make significant changes that require detailed discussions between legal departments at both the central IRB and the relying site IRB. Discussions with some sites are still on-gong with local IRBs at the beginning of the third year of the study. An additional complicating factor in exercise studies with respect to IRB requirements is that at 4 sites, we have been required to have 2 sets of IRB approval. This occurs at sites where the Departments of Neurology and Radiology are governed by different local IRBs than the departments overseeing the implementation of the exercise regimen. We also decided to include sites in Canada to increase the generalizability of our results. This includes the further complication that the Canadian Research Ethics Board (REB) operates under different guidelines and is not overseen by the central IRB.

### Covid-19

On March 9, 2020, the novel *coronavirus* (*COVID*-19) *outbreak* was declared a global *pandemic by the World Health Organization.* All studies of humans that planned to initiate recruitment in and around March 2020 have been affected by COVID-19. SPARX3 was no different. Because there are clear differences in how states in the USA and Canada responded to COVID-19, we made the decision to delay the activation of sites until after March 1, 2021. We estimate that COVID-19 delayed SPARX3 by up to 18 months since some sites were not able to collect data on all people with PD before January 2022. For example, PD by itself was not considered an increased risk if a person caught COVID-19. However, the site would be required to exclude someone with PD if they were over 60, had another chronic condition that elevated their risk of severe COVID-19, and were immune compromised or obese. We were fortunate that the study had not yet started and so there have been no participants that have been unable to maintain their exercise regimen. However, one unexpected consequence of the delay is that some of our potential participants had to wait several months before being screened, and initiated dopaminergic therapy during that waiting period, thus no longer qualifying.

## Conclusion

In summary, SPARX3 will be the first Phase 3 clinical trial of exercise dose in PD. It will answer the question of whether high-intensity exercise differs to moderate-intensity exercise in affecting the rate of disease progression as measured by the MDS-UPDRS motor score (Part III) in early PD, disease duration less than 3 years from diagnosis. In addition, it will also determine if high-intensity exercise differs to moderate-intensity exercise in affecting the SSBR, functional measures of performance, gait, and blood-derived biomarkers, including inflammatory markers. There is an abundance of preclinical evidence, epidemiological evidence, mechanistic evidence, and randomized clinical evidence supporting the beneficial effects of endurance exercise on PD. This will be the first Phase 3 randomized clinical trial designed to test the efficacy of high-intensity exercise compared to moderate-intensity. As a final comment, it is worth noting that one important benefit of exercise that is perhaps underappreciated is that it is accessible to all. Medications have a cost associated with them and require access to physicians with experience in treating Parkinson’s disease. Exercise does not.

## Trial status

At the time of this publication, the study is being conducted under protocol Version Number 1.7 10/18/2021. The study began recruitment in March 2021 with estimated enrollment to be completed July 2025.

## Data Availability

The final de-identified dataset, protocol, data documentation, and statistical code will be shared with NINDS to be deposited in the NINDS Data Repository for sharing with other investigators within 1 year of the primary publication or within 18 months of the study close whichever occurs earliest.

## Abbreviations

AE: Adverse event
BDI: Beck Depression Inventory
BDNF: Brain-derived neurotrophic factor
CDCC: Clinical and Data Coordinating Center
CRF: Case report form
CRP: C-reactive protein
DAT: Dopamine transporter
DSMB: Data and Safety Monitoring Board
EDC: Electronic data capture system
ESC: Executive Steering Committee
GPLD1: Glycosylphosphatidylinositol-specific phospholipase D1
GXT: Maximal graded exercise test
HRmax: Maximum heart rate
IRB: Institutional Review Board
MoCA: Montreal Cognitive Assessment
MDS-UPDRS: Movement Disorders Society-Unified Parkinson’s Disease Rating Scale
Neuro-QOL: Quality of Life in Neurological Disorders
NIH: National Institutes of Health
NINDS: National Institute of Neurologic Disease and Stroke
PD: Parkinson’s disease
PDGene: PD GENEration
PDQ-39: Parkinson’s Disease Questionnaire-39
PI: Principal Investigator
PID: Participant Identification
PIGD: Postural instability gait disorder
PPMI: Parkinson’s Progression Markers Initiative
PSG: Parkinson Study Group
REB: Research Ethics Board
RER: Respiratory exchange ratio
SAE: Serious adverse event
SAP: Statistical Analysis Plan
SPARX3: Study in PARkinson’s disease of eXercise Phase 3 clinical trial
SPECT: Single Photon Emission Computerized Tomography
SSC: Sub-Steering Committee
SSBR: Striatal specific binding ratios
SWEDD: Scans without evidence of dopamine deficit
TD: Tremor dominant
VO_2peak_: Peak volume of oxygen
